# Supplementary material for: A Cost-Effectiveness Analysis of Newborn Screening for Severe Combined Immunodeficiency in the UK
Source: Int J Neonatal Screen. 2019 Aug 30;5(3):28. doi: 10.3390/ijns5030028 (PMC7510246; doi:10.3390/ijns5030028)
Supplement: Supplementary file 1 [file IJNS-05-00028-s001.pdf]

Supplementary Material

Table S1: Detailed cost table

Cost description		Cost 2014/15	Reference
Screening	Band 5 worker (50% FTE)	£12,744	[1]
	Workstation	£2,700	[2]
	Screening test (per baby)	£3.50	[3]
Presumptive positives	Flow cytometry	£25	[3]
	1 x immunology appointment	£251	[4]
	**Total**	**£276**	
Follow up preterm & secondary to other conditions	2 x immunology appointments	£503	[4]
	2 x flow cytometry tests	£50	[3]
	**Total**	**£553**	
Syndromes 4 year follow up	2 x multispecialty appointments per year	£2,011	[4]
	2 x flow cytometry tests per year	£50	[3]
	**Total 4 years (undiscounted)**	**£5,107**	
	**Total 4 years (discounted)**	**£4,872**	
Idiopathic SCID 0-2 years old	3 x immunology appointments per year	£754	[4]
Idiopathic SCID 2-5 years	2 x immunology appointments per year	£503	[4]
Idiopathic SCID (IG and antibiotics)	Immunoglobulin 1st year	£1,789	[5] [6] [7]
	Immunoglobulin 2nd year	£2,716	[5] [6] [7]
	Immunoglobulin 3rd year	£3,319	[5] [6] [7]
	Immunoglobulin 4th year	£3,875	[5] [6] [7]
	Immunoglobulin 5th year	£4,253	[5] [6] [7]

	Antibiotics 1st year	£310	[5] [6]
	Antibiotics 2nd year	£620	[5]
	Antibiotics 3rd year	£620	[5]
	Antibiotics 4th year	£620	[5]
	Antibiotics 5th year	£620	[5]
	**Total 5 years (undiscounted)**	**£21,757**	
	**Total 5 years (discounted)**	**£20,142**	
Diagnosis SCID,	1 x immunology appointment	£251	[4]
	1 x genetic test	£567.5	[8]
	**Total**	**£711**	
Diagnosis idiopathic SCID	1 x immunology appointment	£251	[4]
	1 x genetic test (206 exome panel)	£1,300	[8]
	**Total**	**£1,551**	
Diagnosis syndromes	1 x immunology appointment	£251	[4]
	50% 1 x genetic test (206 exome panel)	£1,300	[8]
	**Total**	**£1,551**	
Enzyme Replacement Therapy (ERT) (Adagen)*	ERT 1 vial per week	£7,500	[9]
	Administration – 1 x non clinical immunology appointments per week	£180	[4]
	**Early diagnosed – 11 weeks**	**£84,475**	
	**Late diagnosed - 26 weeks**	**£199,668**	
Inpatient Care	Day cost inpatient paediatric disorder of the immunity (average)	£1,495	[4]
	Day cost inpatient paediatric critical care level 3	£1,967	[4]
HSCT	Cost of HSCT – 54 days	£80,556	[4]

	**Early diagnosed HSCT – HSCT + 29 days non-critical care + 2.6 days critical care**	**£128,363**	[3, 4]
	**Late diagnosed HSCT – HSCT + 90 days non-critical care + 3.8 days critical care**	**£231,186**	[3, 4]
Gene therapy (GT)	Cost of Strimvelis	£509,027	[10]
	**Early diagnosed GT – GT + 12 days non-critical care + 0.25 days critical care**	**£527,829**	[3, 4]
	**Late diagnosed GT – GT + 45 days non-critical care + 3.3 days critical care**	**£585,994**	[3, 4]
**Death before transplant**	**12.5 days non-critical care + 12.5 days critical care⁺**	**£43,368**	[3, 4]
Follow up SCID well 1st year	4 x immunology appointments per year	£1,005	[4]
Follow up SCID well 2nd-3rd year	2 x immunology appointments per year	£503	[4]
Follow up SCID well 4th year +	1 x immunology appointments per year	£251	[4]
Follow up SCID not well 1st year	6 x immunology appointments per year	£1,508	[4]
Follow up SCID not well 2nd year	4 x immunology appointments per year	£1,005	[4]
Follow up SCID not well 3rd-4th year	2 x immunology appointments per year	£503	[4]
Follow up SCID not well 5th year+	1 x immunology appointments per year	£251	[4]
SCID – enteral feeding	Gastrostomy surgery	£1,539	[4]
	6 x dietician appointments (per year)	£496	[4]
	Feeds 1st year (50% calorific requirements)	£1,315	[5]

	Feeds 2nd year (50% calorific requirements)	£1,996	[5]
SCID – Mild developmental delay	0-3 years	£1,404	[11] [5]
	4-11 years	£24,138	[11] [5]
	12-17 years	£24,138	[11] [5]
	18+ years	£9,347	[11] [5]
IG	For whole life (cost increases with age up to 18 years)	£1,789-£16,481	[5] [7] [6]
Antibiotics	1st year per year (125 mg per day, oral solution)	£310	[5]
	2nd-5th year per year (250mg per day, oral solution)	£620	[5]
	6th year+ per year (250mg per day, tablets)	£28	[5]
Steroids (2 years)	2 mg per kg per day	£772 - £943	[5] [7]
ADHD (5-18 years)	Medication per year	£295	[5]
	2 x CAHMs appointments per year	£599	[4]

FTE – Full time equivalent, SCID – Severe combined immunodeficiency, IG – Immunoglobulin, ERT – Enzyme replacement therapy, HSCT – Hematopoietic stem cell transplant, GT – Gene therapy, ADHD – Attention deficit hyperactivity disorder, CAHMS – Child and adolescent mental health service, *Based on clinical advice patients with ADA-SCID were assumed to receive enzyme replacement therapy for 11 for those diagnosed early and 26 weeks for those diagnosed later and would receive gene therapy in the absence of a fully matched donor, + For patients that die before HSCT and those not diagnosed, we have assumed an inpatient stay equivalent to half the pre-HSCT inpatient stay of a late diagnosed SCID case. To account for the seriousness of their condition we have estimated that the total inpatient stay of 25 days would be split equally with 12.5 days on critical care and 12.5 days on a standard ward

Table 2. Full parameters table and distributions.

Parameter	Mean (95% Confidence Interval)	Distributions for use in the probabilistic sensitivity analysis	Reference
Number of births (UK)	780835	N/A	[12-14]
Incidence of SCID	1:49,000 (1:39,857, 1:61,527)	Beta (82; 4,012,522)	[15]
Incidence of undiagnosed SCID	1:521,000 (1:167,052, 1:7,236,800)	Beta (1.5; 780,833)	[15]
Incidence of syndromes	1:45,000 (1:24,390, 1:110,606)	Beta (7.2; 325,208)*	[16]
Incidence of secondary conditions	1:130,000 (1:50,686, 1:782,506)	Beta (2.5; 325,213)*	[16]
Incidence of idiopathic TCL	1:99,000 (1:42,255, 1:432,482)	Beta (3.3; 325,212)*	[16]
Incidence of preterms	1:99,000 (1:42,255, 1:432,482)	Beta (3.3; 325,212)*	[16]
Sensitivity for SCID	0.99 (0.985, 0.998)	Beta (1567.17; 15.83)	[17]
Presumptive positives (20 copies/μl)	0.041% (0.0035%, 0.1018%)	Beta (2.096; 5079)	[18]
Proportion of SCID patients with a family history	0.30 (0.21, 0.41)	Beta (25; 57)	[15]
Proportion of SCID ADA-SCID	0.17 (0.1, 0.26)	Beta (14; 82)	[15]
Proportion of SCID patients with a matched family donor available	0.25 (0.07, 0.5)	Beta (3.5,10.5)	[15]
Pre HSCT mortality (late diagnosed)	0.35	Beta (17; 31)	[19]

Pre HSCT mortality odds ratio (early diagnosed)	0.03	Lognormal (-4.03; 1.05)	[19]
HSCT mortality (late diagnosed)	0.39	Beta (12; 19)	[19]
HSCT mortality odds ratio (early diagnosed)	0.15	Lognormal (-2.1; 0.6)	[19]
ADA-SCID pre HSCT morality (late diagnosed)	0.21	Beta (10, 38)	[3, 19]
ADA-SCID pre HSCT mortality odds ratio (early diagnosed)	0.06	Lognormal (-3.31; 1.07)	[19]
ADA-SCID HSCT mortality (matched family donor) (late diagnosed)	0.33	Beta (4; 8)	[19]
ADA-SCID HSCT mortality odds ratio (matched family donor) (early diagnosed)	0.11	Lognormal (-2.91; 1.2)	[19]
ADA-SCID Gene therapy mortality	0.05	Beta (1; 18)	[19]
Number of days HSCT	54.0	N/A	[3]
Early diagnosis - Total days non-critical care	82.6 (50.3, 122.8)	Gamma (19.73; 4.19)	[3]
Early diagnosis - Total days critical care	3.96 (0.15, 8.41)	Gamma (1.35; 1.92)	[3]
Late diagnosis - Total days non-critical care	144 (108.6, 184.3)	Gamma (55.39; 2.6)	[3]
Late diagnosis - Total days critical care	8.19 (3.72, 14.4)	Gamma (8.9; 0.92)	[3]
Early diagnosis - Gene therapy - Total non-critical care preGT	12.25 (7.8, 17.7)	Gamma (22.97; 0.53)	[3]

Early diagnosis - Gene therapy - Total critical care preGT	0.25 (0.03, 0.7)	Gamma (1.94; 0.13)	[3]
Late diagnosis - Gene therapy - Total non-critical care preGT	45.7 (35.6, 57.1)	Gamma (69.72; 0.66)	[3]
Late diagnosis - Gene therapy - Total critical care preGT	4.37 (1.59, 8.53)	Gamma (5.93; 0.74)	[3]
QALYs - early diagnosis	0.95	Beta (212.39; 6.62)	[20]
QALYs - late diagnosis	0.82	Beta (165.35; 21.89)	[20]

UK - United Kingdom, SCID - severe combined immunodeficiency disorder, TCL - T Cell lymphopenia, TREC - T cell receptor excision circle, ADA - adenosine deaminase deficiency, HSCT - hematopoietic stem cell transplantation , QALYs - quality adjusted life years. * The denominator used in the sampling was taken to be 10% of the Californian birth rate in order to take into account the uncertainty with using non UK data.

Table S3: Long-term outcomes

Parameter	Early Diagnosed				Late Diagnosed				Reference
	Proportion	Distribution	Cases	95% CI	Proportion	Distribution	Cases	95% CI	
Requires Immunoglobulin	0.25	Beta (9; 27)	3.926	(1.9,6.7)	0.25	Beta (9; 27)	2.337488	(1.1,4.0)	[21]
Requires immunosuppressive drugs (steroids)	0.056	Beta (4; 67)			0.056	Beta (4; 67)			[22]
Considered healthy	0.88	Beta (36.08; 4.92)	13.85	(10.2,18.1)	0.85	Beta (59.5; 10.5)	8.10096	(5.9,10.6)	[23]
No problems	0.49	Beta (20.09; 20.91)	7.684	(4.9,11.0)	0.29	Beta (20.3; 49.7)	3.597459	(2.4,5.1)	[23]
Requires standing antibiotics	0.25	Beta (10.25; 30.75)	3.932	(2.0,6.5)	0.29	Beta (20.3; 49.7)	2.535932	(1.6,3.7)	[23]
Persistent rashes	0.23	Beta (9.43; 31.57)	3.624	(1.7,6.2)	0.29	Beta (20.3; 49.7)	2.451313	(1.5,3.6)	[23]
ADHD	0.16	Beta (6.56; 34.44)	2.505	(1.0,4.6)	0.17	Beta (11.9; 58.1)	1.547135	(0.9,2.4)	[23]
Diarrhea	0.05	Beta (2.05; 38.95)	0.799	(0.1,2.1)	0.19	Beta (13.3; 56.7)	1.160669	(0.6,1.9)	[23]
Height <3rd percentile	0.05	Beta (2.05; 38.95)	2.343	(1.2,3.9)	0.17	Beta (11.9; 58.1)	1.498138	(0.8,2.5)	[23]
Weight <3rd percentile	0.02	Beta (0.82; 40.18)	0.328	(0.0,1.3)	0.17	Beta (11.9; 58.1)	0.928681	(0.5,1.6)	[23]
Warts	0.11	Beta (4.51; 36.49)	1.724	(0.5,3.6)	0.16	Beta (11.2; 58.8)	1.276165	(0.7,2.1)	[23]
Asthma	0.15	Beta (6.15; 34.85)	2.363	(0.9,4.5)	0.16	Beta (11.2; 58.8)	1.456902	(0.8,2.3)	[23]
Developmental delay	0.05	Beta (2.05; 38.95)	0.793	(0.1,2.2)	0.18	Beta (12.6; 57.4)	1.111199	(0.6,1.9)	[23]
GERD	0.05	Beta (2.05; 38.95)	0.782	(0.1,2.1)	0.04	Beta (2.8; 67.2)	0.41712	(0.1,0.9)	[23]
Oral aversion	0.02	Beta (0.82; 40.18)	0.313	(0.0,1.2)	0.04	Beta (2.8; 67.2)	0.28656	(0.1,0.7)	[23]
Hyperthyroidism	0.03	Beta (1.23; 39.77)	0.471	(0.0,1.6)	0.01	Beta (0.7; 69.3)	0.18217	(0.0,0.5)	[23]
Seizure disorder	0.02	Beta (0.82; 40.18)	0.313	(0.0,1.2)	0.01	Beta (0.7; 69.3)	0.138039	(0.0,0.4)	[23]
Skin GvHD	0.04	Beta (4.44; 106.56)	0.63	(0.2,1.4)	0.04	Beta (4.44; 106.56)	0.375435	(0.1,0.8)	[23]
Cerebral palsy	0.02	Beta (2.22; 108.78)	0.318	(0.0,0.9)	0.02	Beta (2.22; 108.78)	0.189226	(0.0,0.5)	[23]
Autoimmune disease	0.02	Beta (2.22; 108.78)	0.313	(0.0,0.8)	0.02	Beta (2.22; 108.78)	0.186521	(0.0,0.5)	[23]

CI – Confidence Interval, ADHD –Attention deficit hyperactivity disorder, GERD – Gastroesophageal reflux disease, GVHD – Graft versus host disease

Figure S1: QALY dimensions

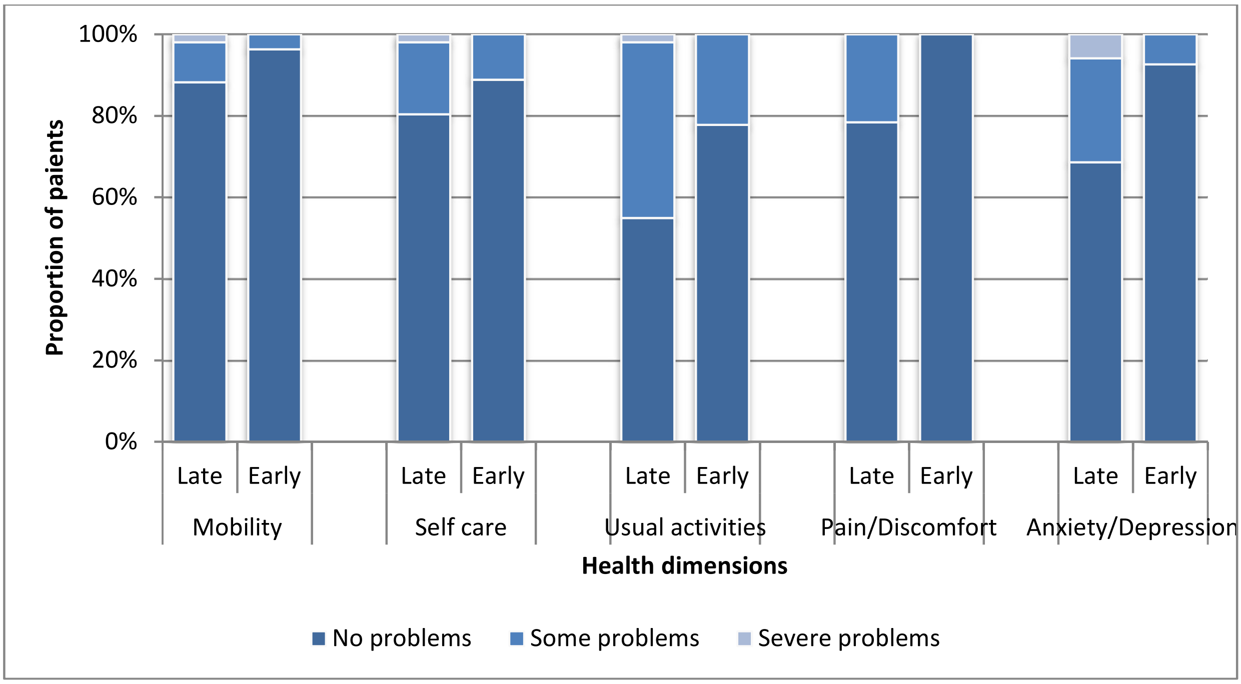

The EQ5D assessments for each dimension are presented in Figure E1 for the diagnosed at birth cohort (early) and the diagnosed later cohort (late). They demonstrate a consistent impact of early diagnosis across all dimensions.

Table S4: QALY decrement threshold analysis for false positive cases and those with non-SCID TCL who are healthy at birth

	Cost per TRECT test £3.50					
	Discounting 3.5% (costs and QALYs)			Discounting 1.5% (costs and QALYs)		
	Mean	95% confidence interval		Mean	95% confidence interval	
Baseline cost effectiveness	£18,588	£12,220	£28,661	£12,090	£7,945	£17,935
Baseline incremental cost	£3,337,979	£2,356,494	£4,480,687	£3,797,777	£2,564,260	£5,324,277
Baseline QALYs gained	180	114	271	314	202	464
Excess QALYs before £20,000 threshold is crossed	13	-4	47	124	74	197
Excess QALYs before £30,000 threshold is crossed	68	35	122	188	117	286
Non-SCID TCL health at birth analysis						
Number of newborns with non-SCID TCL healthy at birth	8	3	17	8	3	17
Minimum QALY decrement for those healthy at birth to increase the screening programme cost effectiveness to be:						
greater than £20,000 threshold	1.5	-1.3	2.7	14.8	25.2	11.4
greater than £30,000 threshold	8.1	12.1	7.0	22.4	39.8	16.5
False positive analysis						
False positives	266	24	796	266	24	796
Minimum QALY decrement for those healthy at birth to increase the screening programme cost effectiveness to be:						
greater than £20,000 threshold	0.05	-0.16	0.06	0.47	3.05	0.25
greater than £30,000 threshold	0.26	1.46	0.15	0.71	4.81	0.36

TREC – T-cell receptor excision circles, QALYs – quality adjusted life years, TCL – T-cell lymphopenia

References

1. Curtis LB, Burns A. Unit Costs of Health and Social Care 2015. Canterbury; 2015. Available at: https://www.pssru.ac.uk/project-pages/unit-costs/. Accessed February 17th 2016

2. PCR UV Laboratory Cabinets. Camlab; 2016. Available at: http://www.camlab.co.uk/uv-cabinet-timer-0-24hrs-p29495.aspx. Accessed February 17th 2016

3. Gaspar B (University College London - Great Ormond Street Institute of Child Health, London, UK) Personal Communication – Length of stay and other costs estimates. 2016.

4. NHS Reference costs 2014-15. Department of Health and Social Care; 2015. Available at: https://www.gov.uk/government/publications/nhs-reference-costs-2014-to-2015. Accessed February 17th 2016

5. Paediatric Formulary Committee. *BNF for Children* (online) London: BMJ Group, Pharmaceutical Press, and RCPCH Publications. Available at: http://www.medicinescomplete.com. Accessed February 17th 2016

6. Liu Z, Albon E, Hyde C. The effectiveness and cost-effectiveness of immunoglobulin replacement thearpy for primary immunodeficiency and chronic lymphocytic leukaemia: a systmatic review and economic evaluation. West Midlands Health Technology Assessment Collaboration, Department of Public Health and Epidemiology, The University of Birmingham; 2005. Available at: https://www.birmingham.ac.uk/Documents/collegemds/haps/projects/WMHTAC/REPreports/2005/IgRT.pdf. Accessed February 17th 2016

7. Health Survey for England 2011 Children trend tables. The Health and Social Care Information Centre; 2012. Available at: https://digital.nhs.uk/data-and-information/publications/statistical/health-survey-for-england. Accessed February 17th 2016

8. Find a Test. National Health Service UK Genetic Testing Network; 2016. Available at: http://ukgtn.nhs.uk/find-a-test/. Accessed February 17th 2016

9. Leadiant BioSciences. Personal Communication - Price of Adagen in the UK. 2016

10. Why gene-therapy drugs are so expensive. The Economist. 2016. Available at: https://www.economist.com/the-economist-explains/2016/08/04/why-gene-therapy-drugs-are-so-expensive. Accessed February 23rd 2017

11. Chilcott J, Bessey A, Pandor A, Paisley S. Expanded newborn screening for inborn errors of the metabolism, Health Economics, Report for consultation. 2013. Available at: https://legacyscreening.phe.org.uk/ga1. Accessed February 17th 2016

12. Live births, 1887 to 2014. Northern Ireland Statistics and Research Agency; 2015. Available at: https://www.nisra.gov.uk/publications/birth-statistics. Accessed November 15th 2016

13. Vital Events - Births. National Records of Scotland; 2015. Available at: https://www.nrscotland.gov.uk/statistics-and-data/statistics/statistics-by-theme/vital-events/general-publications/vital-events-reference-tables. Accessed November 15th 2016

14. Birth Summary Tables - England and Wales 2014. Office for National Statistics; 2015. Available at: https://www.ons.gov.uk/peoplepopulationandcommunity/birthsdeathsandmarriages/livebirths/bulletins/birthsummarytablesenglandandwales/2017. Accessed November 15th 2016

15. Gaspar B (University College London - Great Ormond Street Institute of Child Health, London, UK) Personal Communication – UK incidence estimates. 2015.

16. Amatuni GS, Currier RJ, Church JA, Bishop T, Grimbacher E, Nguyen AA-C, et al. Newborn Screening for Severe Combined Immunodeficiency and T-cell Lymphopenia in California, 2010–2017. Pediatrics. 2019;143(2). https://doi.org.10.1542/peds.2018-2300

17. van der Spek J, Groenwold RH, van der Burg M, van Montfrans JM. TREC Based Newborn Screening for Severe Combined Immunodeficiency Disease: A Systematic Review. J Clin Immunol. 2015;35(4):416-30. https://doi.org/10.1007/s10875-015-0152-6

18. Adams SP, Rashid S, Premachandra T, Harvey K, Ifederu A, Wilson MC, et al. Screening of neonatal UK dried blood spots using a duplex TREC screening assay. Journal of Clinical Immunology. 2014;34(3):323-30. https://doi.org/10.1007/s10875-014-0007-6

19. Brown L, Xu-Bayford J, Allwood Z, Slatter M, Cant A, Davies EG, et al. Neonatal diagnosis of severe combined immunodeficiency leads to significantly improved survival outcome: The case for newborn screening. Blood. 2011;117(11):3243-3246. https://doi.org/10.1182/blood-2010-08-300384

20. Gaspar B; Ladomenou F (University College London - Great Ormond Street Institute of Child Health, London, UK) Personal Communication – EQ-5D-3L estimates. 2016

21. Slatter MA, Brigham K, Dickinson AM, Harvey HL, Barge D, Jackson A, et al. Long-term immune reconstitution after anti-CD52-treated or anti-CD34-treated hematopoietic stem cell transplantation for severe T-lymphocyte immunodeficiency. J Allergy Clin Immunol. 2008;121(2):361-7. https://doi.org/10.1016/j.jaci.2007.10.035.

22. Neven B, Leroy S, Decaluwe H, Le Deist F, Picard C, Moshous D, et al. Long-term outcome after hematopoietic stem cell transplantation of a single-center cohort of 90 patients with severe combined immunodeficiency. Blood. 2009;113(17):4114-24. https://doi.org/10.1182/blood-2008-09-177923

23. Railey MD, Lokhnygina Y, Buckley RH. Long-term clinical outcome of patients with severe combined immunodeficiency who received related donor bone marrow transplants without pretransplant chemotherapy or post-transplant GVHD prophylaxis. J Pediatr. 2009;155(6):834-40. https://doi.org/10.1016/j.jpeds.2009.07.049
